# The role of genome composition and activation in shaping the translocation landscape in health and disease

**DOI:** 10.18103/mra.v12i12.6212

**Published:** 2024-12-29

**Authors:** Anna Oncins, Jessica Velten, Renée Beekman

**Affiliations:** 1 Centre for Genomic Regulation (CRG), Barcelona Institute of Science and Technology (BIST), Barcelona, Spain; 2 Universitat Pompeu Fabra (UPF), Barcelona, Spain; 3 Institut d’Investigacions Biomèdiques August Pi i Sunyer (IDIBAPS), Barcelona, Spain

**Keywords:** Translocations, Genome Composition, Epigenetic Landscapes, Hematological Malignancies

## Abstract

Translocations are rearrangements produced upon erroneous repair of double-strand breaks, fusing segments of non-homologous chromosomes. These events can cause chimeric protein expression and other transcriptional alterations, eventually driving oncogenic transformation. Despite their significance, the factors shaping the heterogeneous translocation landscape in healthy individuals and cancer patients remain incompletely understood. In this review, we focus on genomic content and activation as two fundamental factors associated with translocation formation and selection. While emphasizing the critical role of double-strand breaks and interchromosomal contacts in translocation formation, we discuss that selective advantage is likely the main driver shaping translocation landscapes in health and disease. Finally, we address that it remains difficult to disentangle the effect of translocation formation from the influence of selective pressure, and point out that unraveling their separate contribution in future studies will be key to better understand early tumorigenesis.

## 1 Introduction

Translocations are structural variants (SVs) characterized by the exchange of large chromosomal fragments. Although translocations have been observed in healthy individuals^1–3^, they are usually associated with oncogenic development, seen among others in a large fraction of hematological malignancies^4,5^. Conceptually, every genomic region can be affected by a translocation. However different lines of evidence show that translocation landscapes do not follow random distributions. Early modeling strategies, for example, already demonstrated that combinatorial patterns of chromosomal exchange rates are different from those estimated under the assumption that all chromosomes fuse with equal likelihood^6^. Moreover, translocations found in tumors do not uniformly affect the genome but tend to concentrate at specific genomic sites, targeting only a small set of genes. This unequal representation has at least two underlying causes, namely differences in the probability of translocation formation at different genomic regions, and downstream selection of the formed translocations dependent on the fitness effect that they exert. To gain a comprehensive view of the origin of heterogeneity of oncogenic translocations, we review the effects of genomic and epigenomic properties on translocation formation and translocation selection, using hematological malignancies as a model. More specifically, we discuss the role of genome composition and activation during translocation formation and maintenance in the context of selective pressure, and underline the gaps of knowledge regarding translocation formation probabilities. Lastly, we address that cell-type specific genome activation has a strong influence on the fitness effects of translocations, whereby cells carrying translocations that increase fitness experience a selective advantage that could finally lead to malignancy. Overall, we provide a holistic genomic and epigenomic-oriented perspective of the possibilities and constraints that shape the translocation landscape in malignancies.

## 2 Genomic and epigenomic characteristics that relate to translocation formation

**Figure 1:**
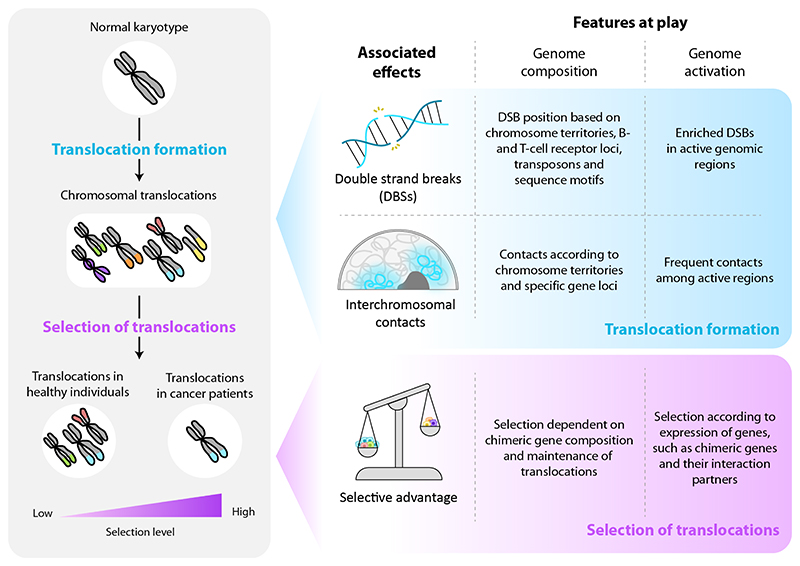
Translocation landscapes in health and disease. The evolution from a normal karyotype to the landscape of translocations, with gradually increasing levels of selective pressure from healthy individuals to cancer patients (left), is influenced by genomic composition and activation that closely relate to formation and selection of translocations (right).

Translocations are formed upon the erroneous repair of double-strand breaks (DSBs) that can be induced under physiological circumstances, for example during immune cell development, or as a consequence of DNA damage caused by exogenous or endogenous carcinogens, such as UV light and reactive oxygen species respectively. Apart from DSBs, the physical proximity of interchromosomal loci is key to allow broken segments to fuse. Importantly, whether DSBs occur before or after two loci get close in 3D space remains controversial. Two exclusive models have been proposed in this respect, the “breakage-first” and “contact-first” models^7^. In the first model, genomic regions with DSBs are proposed to move towards each other, increasing their probability of forming translocations. This model could explain that in different hematological cell lines, the *MLL* and *AF4* loci are further away in 3D space than the *MLL* and *ENL* loci, while MLL::AF4 translocations are more frequently found in hematological tumors^8^. However, we have to keep in mind that other driving forces, such as distinct selective advantages in the presence of the MLL::AF4 and MLL::ENL fusion proteins, could also play a role in establishing these differences. The “contact-first” model, on the other hand, states that translocations preferentially occur between regions that are already in close spatial proximity prior to DSB formation. This hypothesis is based on numerous studies suggesting a higher proximity between genes that are usually translocated, such as *MYC* and *IGH*, in healthy lymphocytes^9^. In the following sections, we do not go further into the link between DSBs and interchromosomal interactions, but we address how genome composition and genome activation are linked to each of these parameters independently (Figure 1).

### 2.1 Genomic Features Are Associated With Translocation Formation

The composition of the human genome determines where genes and other genetic elements are located, their relative position to each other, and their placement up- or downstream of the centromere, as well as chromosomal characteristics such as their length, gene density or GC content. Furthermore, the distribution of chromosomal DNA determines where specific sequence motifs reside. All these genomic features impact translocation formation probabilities at different levels, influencing the location of DSBs prior to the generation of translocations and proximities between interchromosomal loci.

First, the presence of DSBs can be influenced by the relative position that chromosomes occupy within the nucleus. At the largest scale of genome organization, chromosomes occupy distinct locations within the nucleus, known as chromosome territories (CTs)^10^. The arrangement of CTs displays a non-random radial organization that correlates with chromosomal features. For instance, gene-rich, short chromosomes with high GC content tend to be located in the nuclear center, while gene-poor, large chromosomes with low GC content are more often found in the nuclear periphery^11–14^. This radial distribution has been hypothesized to act as a protective shield, preventing UV light and other mutagens from damaging gene-rich chromosomes in the nuclear center^15,16^. According to the “bodyguard hypothesis”, which was already postulated in 1975, DSBs are more prone to occur in the nuclear periphery rather than in the center. However, it remains controversial, as more recent studies demonstrated that oxidative and UV damage affect the nuclear center more than the periphery^17^. These results agree with other publications showing that mutation rates positively correlate with local GC content, which tends to concentrate in the inner part of the nucleus^18^. Hence, while it is clear that the radial positioning of chromosomes is associated with a non-random distribution of DSBs, potentially influencing translocation generation, its actual effect remains to be elucidated.

In addition, various sites within the genome rearrange their genomic sequence through DSB and repair under physiological conditions. More specifically, the regions harbouring the B- and T-cell receptor (BCR and TCR) loci need to be rearranged to ensure that the genome of each B and T cell encodes a different BCR or TCR, altogether determining our immune repertoire^19,20^. This process is mediated by the recombination-activating genes 1 and 2 (RAGs) and activation-induced cytidine deaminase (AID)^21,22^. Interestingly, when looking at B-cell-related translocations, AID and RAG motifs are not only found at the breakpoint of the *BCR* locus but they also form hotspots on its chromosomal translocation partners, further underlining the role of these proteins in translocation formation^23–26^. Increased tendencies to generate DSBs make the *BCR* and *TCR* loci prone to form SV hotspots. Indeed, in healthy B cells, the most frequently observed SVs are those involving the B-cell receptor regions, also known as immunoglobulin (IG) loci^3^. By a similar mechanism, genomic regions flanking transposons are also susceptible to suffer DSBs, which are catalyzed by different families of transposases^27–29^. The consequences of these rearrangements go far beyond the simple excision and insertion of DNA, and can be associated with SVs such as inversions, deletions, duplications and translocations^30–33^.

Apart from DSB formation, genome composition also largely associates with interchromosomal interaction events, which set the second requisite for translocation formation. First of all, the radial organization of CTs outlined above greatly impacts the interaction probabilities of different chromosomes, with interactions between gene-rich, short chromosomes with high GC content being the most probable. Moreover, the genome contains loci predisposed to form interchromosomal nuclear DNA hubs, such as nucleoli^34^, which could favour translocation formation among the affected chromosomes. Nucleoli specifically bring genes encoding ribosomal RNA, also known as rDNA loci, close together in 3D space. These rDNA loci are located on the short arms of the acrocentric chromosomes 13, 14, 15, 21 and 22 in the human genome^35,36^. Importantly, however, we did not find any evidence that translocations occur more than by chance among acrocentric chromosomes, or among short gene-rich chromosomes in hematological tumors. Two studies underscore this finding, showing that no proximity-based biases were observed at the interchromosomal level, suggesting a minimal role of this phenomenon in affecting probabilities for translocation formation^23,24^.

### 2.2 Active Genomic Regions Are Prone To Form Translocations

Active regulatory elements, mainly consisting of active enhancers and promoters, demarcate the genome activation landscape. They are part of the broader, cell-type-specific epigenetic landscape, which allows for the diversification of cell types from a single underlying genome. Translocations have been shown to associate with active genomic regions, which could explain their cell-type specificity. Therefore, in this section, we focus on the potential influence of genome activation on the probability of translocation formation, emphasizing the contribution of the active genome to DSB formation and interchromosomal proximity.

Different studies in lymphocytes have shown that translocations are preferentially formed at active transcriptional start sites, even in the absence of RAG and AID^23,24^. Hence, beyond RAG and AID activity, other mechanisms that drive DSBs at active genomic regions must be at play. Different technologies have been developed to study this in further detail, such as DSB-seq and BLESS-seq^37,38^. For example, BLESS-seq experiments in HeLa cells exposed to replicative stress showed an enrichment of DSBs in actively transcribed regions, potentially as a result of colliding transcription and replication forks. Interestingly, genes frequently altered in cancer, including those targeted by translocations, were overrepresented within the affected transcribed regions^38^. Furthermore, DSB-seq analyses showed that active promoters are hotspots of DSBs, due to high levels of Topoisomerase II-induced torsional stress at these loci^37^. Following this trend, genome activation also increases the probability of DSB formation at transposons, favouring their excision. While transposons are normally repressed, the reduction of DNA methylation, as well as the loss of repressive and the gain of activating histone marks, have been linked to their derepression, indicating a crucial role of the epigenetic landscape in controlling transposon activity levels and excision rates^39,40^. Overall, these results suggest that increased DSB formation at active regions could contribute to the observed pattern that translocations are more frequent at active loci.

Beyond its potential role in DSB formation, genome activation is also clearly associated with the radial organization of genomic loci within the nucleus. More specifically, it is generally accepted that active genomic regions reside in the nuclear interior, while inactive sites tend to be closer to the nuclear periphery^41,42^. However, the precise mechanisms by which the causal relationship between 3D organization and genome activity is established remains challenging to define. For instance, it remains unclear whether activity precedes 3D organization or vice versa, mainly due to contradictory studies as well as technical limitations. Importantly, recent developments such as the CRISPR-GO technology provide accurate new tools to create a better understanding of this causal relationship^43^. For instance, CRISPR-GO-induced manipulation of radial positioning towards the nuclear lamina led to decreased expression of some genes, while other loci did not alter their expression. This indicates that only some genes are susceptible to change their activity based on their nuclear positioning. In contrast, another study has shown that genes such as *CFTR* move to the nuclear interior upon transcription activation, showing that nuclear repositioning can be a downstream effect of gene activation^44^. Overall these results indicate that, depending on the gene locus and the cell type, some genes alter their expression before and others after nuclear repositioning, while neither of these relationships exist for many other genes. Importantly, independent of whether genome activation precedes or follows nuclear positioning, active regions tend to be closer to each other within the nuclear interior. This likely explains the observed cell-type specific proximities between active genes that are usually found at translocation breakpoints, such as *MYC* and *IGH*, or *BCL2* and *IGH* in healthy lymphocytes^9^. Overall, these activity-based proximities lead to a higher chance of active regions to physically interact, resulting in a higher probability of translocation formation. Moreover, beyond global interactions of active genomic regions, transcription factor (TF)-based chromatin hubs, also known as transcription factories, drive non-random spatial proximities that might influence the probability of generating translocations^34,45^. Hence, the evidence outlined above provides a plausible link between translocation occurrence and genomic activity, based on induction of interchromosomal proximities.

Overall, in this section we have addressed how genomic and epigenomic features relate to DSB formation and interchromosomal interaction frequencies at different genomic loci, in order to better understand translocation formation probabilities. We furthermore discussed the complexity of proving causal relationships and time-wise dependencies between different features, especially regarding chromosomal characteristics, genome activation and radial positioning. In addition, it is crucial to highlight that multiple factors described above may act simultaneously. One example is the potential combinatorial effect of the “bodyguard hypothesis” and genome activation, favouring DSBs in the nuclear periphery and the nuclear center respectively. Mixtures of these different mechanisms may underlie the final DSB landscape, making it difficult to disentangle the contribution of each individual mechanism to translocation formation. Another example relates to transcription factories in which different genomic regions with the same TF binding site come together upon activation by TFs. Hence, while the distribution of motifs along the linear sequence is important to establish interchromosomal contacts in the context of transcription factories, this becomes only relevant in the presence of the corresponding TFs. Furthermore, we would like to stress that these and other genome-activation based effects introduce an important level of heterogeneity in translocation formation probabilities due to their cell-type specific nature, which needs to be accounted for when analysing translocation landscapes. Finally, we did not find any clear evidence that chromosomal proximities affect the global translocation landscape in hematological malignancies. Hence, such proximities alone are not sufficient to explain the combinations of chromosomal partners observed in oncogenic translocation landscapes. A very likely explanation is that locus-specific activity based proximities and selective pressure play a more dominant role in this context.

## 3 Selection of translocations depends on genomic and epigenomic features

As for many genetic alterations, translocations can induce downstream changes that affect the cellular fitness landscape. This can result in positive or negative selection, due to increased or decreased fitness respectively. Most translocations likely do not exert any change in fitness, though, being so-called neutral alterations. Nevertheless, translocations that do alter cellular fitness play a key role in shaping the translocation landscape both in healthy individuals and in cancer patients. In healthy individuals, these effects will lead to the elimination of cells carrying translocations that exert negative selective pressure, while providing small proliferative advantages to those cells with subtle increases in fitness. In contrast, in the context of tumorigenesis, large increases in fitness are essential to provide the selective advantage needed to develop full-blown tumors. As selective pressure in this context strongly depends on aberrations that healthy cells acquire along their journey to malignancy, oncogenic translocations frequently found in tumors likely provide a strong selective advantage. In this paragraph, we address how genomic and epigenomic features globally influence the selective advantage exerted by translocations, in order to better understand translocation landscape composition in health and disease (Figure 1).

### 3.1 Genomic Features Largely Constrain The Occurrence Of Translocations With Selective Advantage

The genomic sequence determines the linear distance between genes, their position on the positive or negative strand, and their location up- or downstream of the centromere on the small (p) or long (q) arm of the chromosome respectively. Furthermore, the linear DNA sequence forms the basis for the distribution of functional domains along proteins, defining the position of these domains relative to each other, and with respect to the N- or C-terminus of the protein. All these features demarcate the possibilities and constraints to generate translocations that can induce increased cellular fitness.

First, translocations alter the linear DNA sequence, forming chimeric or fusion proteins, as a possible major consequence. Examples in this respect are BCR::ABL and PML::RAR found in chronic myeloid leukemia (CML) and acute promyelocytic leukemia (APL) respectively^46,47^. These chimeric proteins will exhibit new properties depending on the combination of the functional domains of the two individual proteins. Importantly, while in theory many different chimeric proteins can be formed upon translocation formation, the functional domain composition of observed chimeric proteins is different from that expected by chance^48–50^. Globally, DNA binding domains (DBDs), protein interaction domains (PIDs) and kinase domains (KDs), among others, are significantly overrepresented in chimeric proteins^49^. In addition, in hematological tumors, chimeric proteins show enrichment of specific domain combinations such as the PID of one and the histone modification domain (HMD) of another protein, or the KDs, or HMDs of two different proteins^48,50^. Interestingly, in the current era of synthetic biology, an endless number of chimeric proteins, comprising specific domain combinations in any given order, could be engineered and tested for their oncogenic potential. However, the majority of these random products can never be formed by the fusion of two chromosomes in the human genome, as in the cellular context chimeric protein composition is restricted by the fusion of sequences encoding the N-terminal part of one and the C-terminal part of another protein. Hence, the linear order of functional domains of individual proteins, which is dictated by our DNA sequence, determines which chimeric proteins can be formed in a cell, and defines whether they can exert increased fitness effects with the potential to drive oncogenesis. Of note, the formation of a chimeric protein by a translocation is not a stand-alone effect; fusion of the 5’ and 3’ ends of two translocation partner genes (5'TPG and 3'TPG respectively) also results in fusion of their remaining 3’ and 5’ parts on the reciprocal translocation derivative. In addition, both affected genes lose a wild-type copy, resulting in haploinsufficiency. Altogether, these extra events may result in additional, potentially synergistic effects with oncogenic potential. Indeed, in some cases, the reciprocal chimeric gene product has been shown to play a role in tumorigenesis, as shown for the AF4::MLL protein in leukemia^51,52^.

In addition, at the chromosome-wide scale, centromeric positioning is an important genomic parameter to address. Centromeres determine whether chromosomal translocation products, also known as derivatives, can persist throughout cell division. More specifically, translocation derivatives without centromeres will be lost upon mitosis, while derivatives with two centromeres will lead to erroneous cell divisions, likely causing apoptosis. Therefore, only when two centromeres are equally distributed over two derivative chromosomes, they will be retained. This overall restricts the number of translocations that allow the cells to divide properly, which is an essential property to drive selective advantage. Moreover, it has significant implications for the possible existence of fusion genes, as chimeric gene products can only be sustained if formed between genes located on the same strand and the same chromosome arm, or between genes located on different strands and different chromosome arms. Hence, centromeric positioning limits the number of fusion gene combinations that cells can maintain through cell division.

### 3.2 The Genome Activation Landscape Is A Key Determinant For Translocation Selection

Genome activity states influence the gene expression, and thus the selective pressure changes caused by translocations. First of all, fusion genes can only be expressed if the promoter of the 5’TPG is active. This dependency becomes clear in the context of hematological malignancies. The 5’TPGs of oncogenic chimeric genes show high expression levels in blood, allowing the fusion genes formed at these loci to be highly expressed. Furthermore, the cell-type specific expression patterns of 5’TPGs and 3’TPG binding partners explain why fusion genes infer oncogenic potential in a cell-type specific manner^50^. Second, beyond fusion gene formation, translocations can induce the expression of proto-oncogenes if placed in close proximity to active enhancers. Clear examples of gain of proto-oncogene expression are seen in the context of IGH translocations, inducing the expression of TFs located close to the breakpoint, such as MYC, BCL2, BCL6 and CCND1 in non-Hodgkin lymphoma^53^. T-cell acute lymphoblastic leukemias that contain translocations involving the *TCR* locus provide further examples of this phenomenon^54^. Of note, induced proto-oncogene activation depends on other epigenomic features such as boundaries of topologically associated domains (TADs) and other regions with insulating potential. When such boundary elements are present between an active enhancer and a proto-oncogene that are brought together upon translocation formation, this rearrangement will have a limited impact on protooncogene expression^55^. Third, loss of gene expression can occur when a translocation uncouples an active enhancer from its target gene, for example in the case of *GATA2* following the t(3;3)(q21;q26) translocation^56^. This example highlights another case of synergy due to the co-occurrence of gain of proximity to sequences with regulatory potential at one of the derivatives, and the loss of this proximity at the reciprocal translocation product, whereby *EVI1* and *GATA2* are respectively affected^56^. Finally, it should be considered that the gain of proximity to sequences with regulatory potential can affect multiple downstream genes, representing an additional synergistic mechanism.

Altogether, in this section we have highlighted that genome composition and activation strongly affect selective advantage exerted by translocations. While we addressed them individually, the combination of these features has a clear impact on the overall level of selective advantage that influences translocation landscapes. This can for example be appreciated for fusion genes with oncogenic properties based on their linear sequence, which can only exert their oncogenic function if expressed. On the other hand, non-oncogenic fusion genes can be expressed without any consequence. Hence, the genome sequence and activation of chimeric genes together play a key role in their downstream fitness effects. Another key message is that cell-type specificity of genome activation patterns gives a clear explanation for the tumor-type specific nature of translocations. Especially, their oncogenic impact depends on the epigenetic landscape, limiting the number of cell types in which particular translocations can have an oncogenic effect, and thus restricting the tumor types they can induce. Importantly, epigenetic states providing the fertile soil for translocations to thrive do not only relate to cell types, but could also be linked to cell subtypes or caused by genetic alterations other than translocations. Finally, we highlight that translocation-induced alterations in genomic content and/or concurrent coupling and decoupling of regulatory elements and their target genes form the basis for potential synergistic effects that may drive tumorigenesis.

## 4 Conclusions

Translocations in hematological malignancies are largely tumor-type specific and affect particular genomic sites more than others. Within this review, we provided a detailed overview of how genomic and epigenomic features shape the unevenly distributed oncogenic translocation landscape. We considered that both the possibilities and constraints that these features impose were important to address explicitly. Regarding the constraints, we speculate that many potent oncogenic fusions can be generated by random shuffling of protein domains, but the composition of the human genome does not allow these events to occur upon translocation formation, or they get lost upon cell division due to centromeric disbalance in chromosomal derivatives. From here the question arises whether the need to limit the possibilities to form detrimental SVs has shaped our genome distribution throughout evolution. In other words, the distribution of our genome, marked by a specific order of our DNA divided over different chromosomes, may prevent the formation of many harmful SVs that would impose strong negative fitness on our species. Furthermore, we highlight that chromosomal proximities alone are likely not a main driver of translocation formation. Importantly though, to gain further insights into this, more unbiased analyses to study the effect of proximity on translocation formation need to be conducted in systems where selective pressure does not play a role.

Unfortunately, it remains challenging to investigate genome-wide translocation landscapes in the absence of selective pressure, as all biological systems will suffer from this to a certain extent. Of note, while translocation landscapes in healthy tissues are not completely free of selection, they could partially fill this gap of knowledge, providing important clues regarding the parameters that drive their generation and persistence before or during early tumor formation. Finally, we show that selective advantage is likely a dominant factor in shaping the oncogenic translocation landscape. This advantage stands or falls by the probability of translocations to induce gene expression alterations that drive tumorigenesis. This broad scala of genome-wide downstream changes can be mediated by translocation-induced fusion gene and proto-oncogene expression, as well as by haplo-insufficiency or loss of tumor suppressor genes. Hence, the effects of individual translocations tunnel into a combined set of genome-wide epigenetic and gene expression changes, with a single translocation as their common driving force. Altogether, these effects can provide the strong selective advantage needed to favour malignant transformation. Translocations will rapidly disappear after their formation though if providing selective disadvantages, and many neutral ones will stay under the radar, while only few will be found in tumor samples. Therefore, further study of translocation landscapes in the absence of selective pressure, as well as genome-wide translocation effects in the context of pre-existing epigenetic states, will be essential to better understand early steps of tumorigenesis.

## Data Availability

There is no data available.
